# Twelve-Month Metastatic State as a Landmark-Based Prognostic Stratifier in Metastatic Sarcoma

**DOI:** 10.3390/cancers18101622

**Published:** 2026-05-17

**Authors:** Theodoros Loupasis, Janet Ruthenberg, Bettina Vogel, Markus Schärer, Georg Schelling, Philip Heesen, Gabriela Studer, Bruno Fuchs, on behalf of the Swiss Sarcoma Network

**Affiliations:** 1 Department of Orthopaedics and Trauma, LUKS Sarcoma-IPU, University Teaching Hospital LUKS, Spitalstrasse, 6000 Lucerne, Switzerland; 2 Department of Orthopaedics and Trauma, Kantonsspital Winterthur, KSW Sarcoma Center, Brauerstrasse 15, 8401 Winterthur, Switzerland; 3 Swiss Sarcoma Network SSN, LUKS Sarcoma-IPU, University Teaching Hospital LUKS, Spitalstrasse, 6000 Luzern, Switzerland; 4 Schulthess Klinik, Lengghalde 2, 8008 Zürich, Switzerland; 5 Faculty of Medicine, University of Zurich, Raemistrasse 71, 8006 Zurich, Switzerland; 6 Department of Radiation Oncology, LUKS Sarcoma-IPU, University Teaching Hospital LUKS, Spitalstrasse, 6000 Lucerne, Switzerland; 7 Faculty of Health Sciences & Medicine, University of Lucerne, Frohburgstrasse 3, 6002 Luzern, Switzerland

**Keywords:** metastatic sarcoma, post-metastasis survival, 12-month metastatic state, landmark analysis, prognostic stratification, oligometastatic disease, polymetastatic disease, real-world-time data, survival modeling, histologic grade

## Abstract

Background: Prognostic assessment in metastatic sarcoma remains challenging, and baseline variables may not adequately reflect outcome after metastatic onset. We investigated whether the 12-month landmark provides clinically relevant prognostic stratification for post-metastasis survival, while institution was examined as a contextual and adjustment variable rather than as the primary analytic focus. Methods: This retrospective observational study was based on prospectively collected real-world-time clinical data from two sarcoma centers. Overall survival (OS) and post-metastasis survival (PMS) were first analyzed descriptively across the two contributing centers using Kaplan–Meier methods. The principal analysis was a landmark-based prognostic stratification of PMS from 12 months after first metastatic documentation, with patients classified at the landmark as oligometastatic or polymetastatic. Univariable and multivariable Cox regression models were used to assess prognostic associations. Results: A total of 236 patients were included (135 at Institution A and 101 at Institution B). Crude OS was broadly comparable between institutions, whereas PMS showed a numerical separation. In the landmark analysis, metastatic state at 12 months clearly stratified subsequent PMS, with persistent oligometastatic disease associated with more favorable outcome than polymetastatic disease. In the reduced multivariable landmark model, polymetastatic state at 12 months remained the strongest independent predictor of inferior PMS (HR 3.09, 95% CI 1.87–5.09; *p* = 0.00001). High histologic grade also retained independent adverse prognostic significance (G3 vs. G1: HR 5.16, 95% CI 1.59–16.79; *p* = 0.006), whereas institution showed only a non-significant trend after adjustment (HR 0.67, 95% CI 0.42–1.07; *p* = 0.094). Conclusions: In metastatic sarcoma, realized metastatic state at 12 months provides stronger landmark-based prognostic stratification for post-metastasis survival than crude institutional comparison. These findings support a longitudinal state-based prognostic framework in which survival after metastatic onset is more informatively stratified by disease state at the 12-month landmark than by crude center attribution.

## Simple Summary

Metastatic sarcoma does not behave uniformly. Some patients develop widespread metastatic disease early, whereas others remain in a more limited metastatic state for longer periods. In this study, we analyzed real-world data from two sarcoma centers to determine whether the metastatic state at 12 months provides useful prognostic information within the metastatic phase of disease. We found that patients who were polymetastatic at 12 months had substantially worse post-metastasis survival than those who remained oligometastatic, whereas the treating institution was less informative after adjustment. These findings suggest that prognosis in metastatic sarcoma may be better understood using a landmark-based, state-focused approach rather than crude center comparison alone.

## 1. Introduction

Sarcomas comprise a heterogeneous group of mesenchymal malignancies with substantial variation in histology, biologic behavior, treatment sensitivity, and clinical outcome [1,2,3]. In the metastatic setting, prognosis is often poor, but survival remains highly variable [4,5,6]. Some patients develop rapidly progressive systemic dissemination, whereas others experience a more limited metastatic course associated with longer survival [7,8,9,10]. This heterogeneity makes prognostic assessment in metastatic sarcoma particularly challenging and suggests that metastatic disease should not be regarded as a uniform clinical state [11].

Conventional prognostic assessment has largely relied on baseline clinicopathologic variables, including grade, tumor size, histologic subtype, stage, and timing of metastasis [12]. Although these factors remain important, they do not fully reflect how metastatic disease behaves after it has emerged [11]. Prior real-world-time analysis from our group suggested that metastatic sarcoma is jointly structured by timing of onset, organ distribution, and metastatic burden, indicating that metastatic phenotype is better understood as a multidimensional evolving state than as a simple binary event [7,13]. In that preceding analysis, lung-confined and multi-organ trajectories, as well as oligo- versus polymetastatic burden, emerged as clinically meaningful phenotype dimensions rather than merely descriptive findings. Against this background, the distinction between oligometastatic and polymetastatic disease may carry prognostic information beyond baseline categorization alone, particularly if assessed as a longitudinal state rather than a static descriptor [8,14].

A landmark-based prognostic framework is useful in this setting because it moves beyond a crude baseline metastatic yes/no distinction and incorporates the realized early metastatic course into survival analysis [15,16]. By anchoring prognosis to a clinically meaningful timepoint after metastatic onset, such an approach allows post-metastatic outcome to be interpreted in relation to disease state that has already evolved over time rather than solely to baseline classification [17]. At the same time, a landmark design remains an intermediate step between static baseline categorization and fully time-dependent survival modeling, because it does not account for post-landmark treatment changes, repeated metastatic state transitions, or evolving patient health status [18,19]. Building on prior evidence that metastatic sarcoma is shaped by timing, distribution, and metastatic burden, the present study examined whether realized metastatic state at 12 months provides clinically relevant prognostic stratification for post-metastasis survival in a two-institution real-world cohort [13,20,21]. We first described OS and PMS across the two contributing centers as contextual survival analyses and then tested whether oligometastatic versus polymetastatic state at the 12-month landmark more informatively stratified subsequent PMS in adjusted landmark models [22]. Institution was treated as a contextual and adjustment variable rather than as the primary analytic focus [14,17].

The 12-month landmark was selected as a pragmatic operational timepoint intended to balance biologic interpretability and analytic feasibility. Clinically, this interval is long enough to capture whether initially limited metastatic disease remains constrained or progresses to broader dissemination during the early metastatic phase. Methodologically, it also preserves a sufficiently large cohort under observation for interpretable post-landmark survival analysis. Accordingly, 12 months should be understood as a clinically meaningful but not universally validated sarcoma-specific threshold.

## 2. Materials and Methods

### 2.1. Study Design and Setting

**Figure 1:**
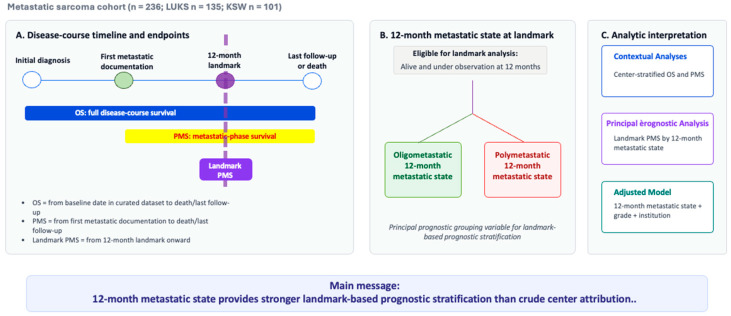
Conceptual framework of landmark-based prognostic stratification by 12-month metastatic state in metastatic sarcoma. The schematic illustrates the analytic structure of the study. Overall survival (OS) was assessed across the full disease course from the baseline date defined in the curated dataset to death or last follow-up. Post-metastasis survival (PMS) was assessed from the first radiologic or histologic documentation of distant metastatic disease to death or last follow-up. For the principal landmark analysis, patients who were alive and under observation at 12 months after first metastatic documentation were stratified according to landmark metastatic status as oligometastatic or polymetastatic, and subsequent PMS was analyzed from the landmark onward. Center-stratified OS and PMS analyses were interpreted as contextual descriptive analyses, whereas the principal inferential analysis focused on whether 12-month metastatic state provides informative landmark-based prognostic stratification for post-metastasis survival.

This study is based on prospectively collected real-world-time clinical data from two sarcoma centers, Institution A and Institution B. The objective was to evaluate whether 12-month metastatic state provides informative landmark-based prognostic stratification for post-metastasis survival in metastatic sarcoma within a harmonized two-institution cohort (Figure 1). The study was not designed as a treatment-comparison or center-ranking analysis.

Clinical data were prospectively documented during routine sarcoma care and subsequently harmonized for analytic use. The curated dataset was analyzed retrospectively according to a predefined statistical framework.

### 2.2. Patient Cohort and Eligibility

Patients were identified from the prospectively maintained sarcoma datasets of the two participating institutions. Eligible patients had documented metastatic (bone and soft tissue) sarcoma and sufficient longitudinal follow-up for survival analysis. Both patients presenting with metastatic disease at diagnosis and patients developing metastatic disease during follow-up were eligible, provided that the timing of metastatic disease and survival status could be determined from the curated dataset.

The descriptive cohort included all eligible patients in the finalized harmonized dataset. For time-to-event analyses, patients were required to have sufficient completeness of the variables relevant to the respective analysis. Accordingly, the evaluable population differed slightly across descriptive analyses, Kaplan–Meier analyses, landmark analyses, and Cox regression models. For the 12-month landmark analysis, patients had to be alive and under observation at 12 months and have an evaluable landmark metastatic status. Patients with missing data in model-defining variables were excluded from the corresponding regression analyses on a complete-case basis.

### 2.3. Variables and Endpoints

Baseline variables extracted from the harmonized dataset included institution, age at diagnosis, sex, histologic subtype, tumor grade, tumor size, timing of metastatic disease, and stage-related variables where available. Histologic grade was analyzed using the available grading categories, with grade 1 (G1) as the reference category in regression analyses.

The principal prognostic variable was 12-month metastatic state, categorized as oligometastatic or polymetastatic and analyzed as the main grouping variable for landmark-based prognostic stratification. Consistent with our preceding triangulation analysis, oligometastatic disease was operationally defined as ≤5 metastatic lesions involving a maximum of two organs, whereas polymetastatic disease was defined as metastatic burden beyond this limit. This classification was assigned at the 12-month landmark after first radiologic or histologic documentation of distant metastatic disease, thereby establishing each patient’s post-landmark burden state. We acknowledge that no universally validated sarcoma-specific oligometastatic cutoff exists and that this threshold should therefore be understood as a pragmatic operational definition rather than as a definitive biologic boundary. It was selected because it was reproducible within the harmonized dataset and conceptually aligned with prior oligometastatic literature, while still requiring disease-specific validation in sarcoma.

This operational classification was based on lesion number and organ count because these variables were reproducibly available across the harmonized dataset. We acknowledge, however, that metastatic severity is broader than burden count alone and may also include dimensions such as tumor volume, site-specific criticality, symptomatic burden, pathologic fracture, neurologic compromise, or brain and other high-risk visceral metastases. These variables were not comprehensively and uniformly captured enough for robust integration into the present landmark framework.

The primary endpoints were overall survival (OS) and post-metastasis survival (PMS). OS was defined as the interval from the baseline date recorded in the curated dataset to death from any cause or last follow-up. PMS was defined as the interval from the first radiologic or histologic documentation of distant metastatic disease to death from any cause or last follow-up.

For the landmark PMS analysis, survival was assessed from the 12-month timepoint after first metastatic documentation, with patients stratified according to the realized metastatic state at 12 months. Patients who had died or were no longer under observation before 12 months were not eligible for this analysis. This design was chosen to evaluate whether 12-month metastatic state provides informative landmark-based prognostic stratification within the metastatic phase of disease. The landmark curves and multivariable model were therefore interpreted as post-metastatic, state-based analyses rather than as full disease-course survival analyses.

The 12-month landmark was chosen as a pragmatic clinically meaningful timepoint intended to capture early metastatic evolution while retaining sufficient patients alive and under observation for interpretable post-landmark analysis. The study was not designed as a formal landmark-optimization exercise comparing alternative timepoints such as 6, 24, or 36 months. Accordingly, 12 months should be interpreted as an operational landmark within the present framework rather than as a definitive biologic cut point for all metastatic sarcomas.

A supportive contextual analysis of AJCC broad stage was also performed for OS. Because this analysis served as contextual validation rather than the principal prognostic analysis, it is best presented in the Supplementary Materials.

### 2.4. Statistical Analysis

Descriptive statistics were used to summarize baseline characteristics for the full cohort and by institution. Continuous variables were summarized using medians and interquartile ranges or other appropriate distribution-based measures, and categorical variables were summarized using counts and percentages. Group comparisons were performed using standard statistical tests according to variable type and distribution.

Survival distributions were estimated using the Kaplan–Meier method. Kaplan–Meier curves were generated for descriptive contextual analyses of OS and PMS across the two contributing centers and for landmark PMS according to the state at the 12-month landmark, which constituted the principal prognostic analysis. OS was additionally explored across AJCC broad stage categories as a secondary contextual analysis.

Center-based Kaplan–Meier curves for OS and PMS were interpreted as descriptive contextual analyses only. The principal inferential analysis used a 12-month landmark design for PMS in order to incorporate early metastatic evolution into prognostic stratification. This approach captures realized disease state up to the landmark, but the present study was not designed to model post-landmark time-varying treatment exposure, repeated metastatic-state transitions, or dynamic health-status covariates. Time-to-event associations were further examined using Cox proportional hazards regression. The proportional hazards assumption was assessed using Schoenfeld residuals. Univariable Cox models were first fitted to assess the association of candidate variables with landmark PMS. A multivariable Cox model was then constructed for the landmark PMS analysis, including institution, tumor grade, and 12-month metastatic state as the principal covariates. An additional multivariable Cox model was fitted for OS, incorporating AJCC broad stage and selected clinicopathologic variables as a contextual secondary analysis.

Hazard ratios (HRs) with 95% confidence intervals (CIs) were reported. All statistical tests were two-sided, and a *p*-value < 0.05 was considered statistically significant. Analyses were performed in R Statistical Software (Version 2026.01.0+392).

Systemic treatment exposure was not modeled as the principal analytic axis of this study; accordingly, the present analyses were not designed to evaluate comparative therapeutic effectiveness. However, we acknowledge that systemic therapy, metastasis-directed surgery, radiotherapy, ablative procedures, and other local consolidative strategies may influence post-metastatic survival and contribute to inter-patient heterogeneity within the metastatic phase.

## 3. Results

### 3.1. Patient Cohort and Baseline Characteristics

A total of 236 patients were included, of whom 135 were treated at Institution A and 101 at Institution B. Baseline characteristics are summarized in Table 1. The median age at diagnosis was lower at Institution A than at Institution B, whereas sex distribution and the major oncologic baseline variables were broadly comparable between institutions. The cohort was histologically heterogeneous, with a predominance of high-grade disease. Overall, the baseline distribution did not suggest a major structural imbalance that would, by itself, explain survival differences at the institutional level.

### 3.2. Contextual Survival Analyses Across the Two Contributing Centers

**Figure 2:**
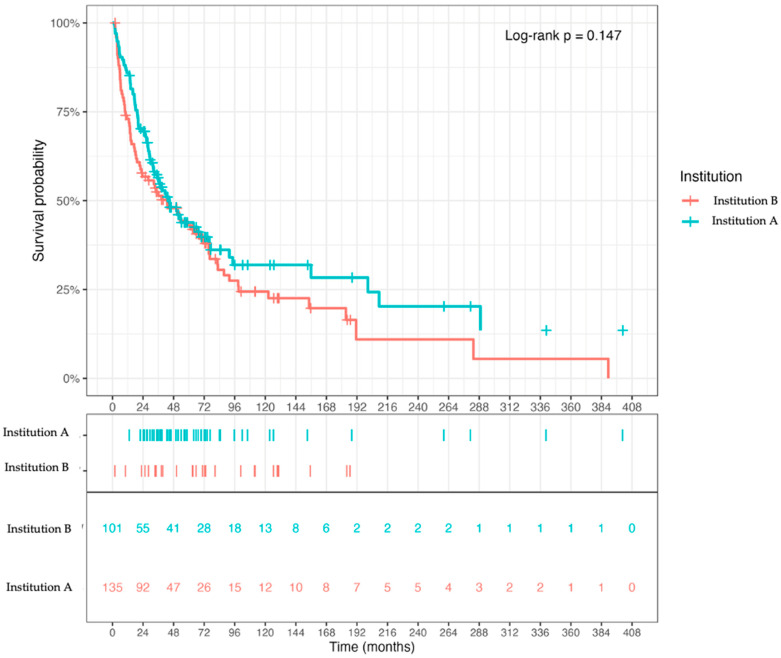
Overall survival by institution. Kaplan–Meier curves showing overall survival (OS) for patients treated at Institution A and Institution B. OS was analyzed as descriptive center-level context across the full disease course.

As a contextual survival analysis, Kaplan–Meier estimation of overall survival (OS) showed largely overlapping survival trajectories across the two contributing centers (Figure 2), indicating that crude OS was broadly comparable at the center level. Median OS was 43.0 months at Institution B and 44.8 months at Institution A.

**Figure 3:**
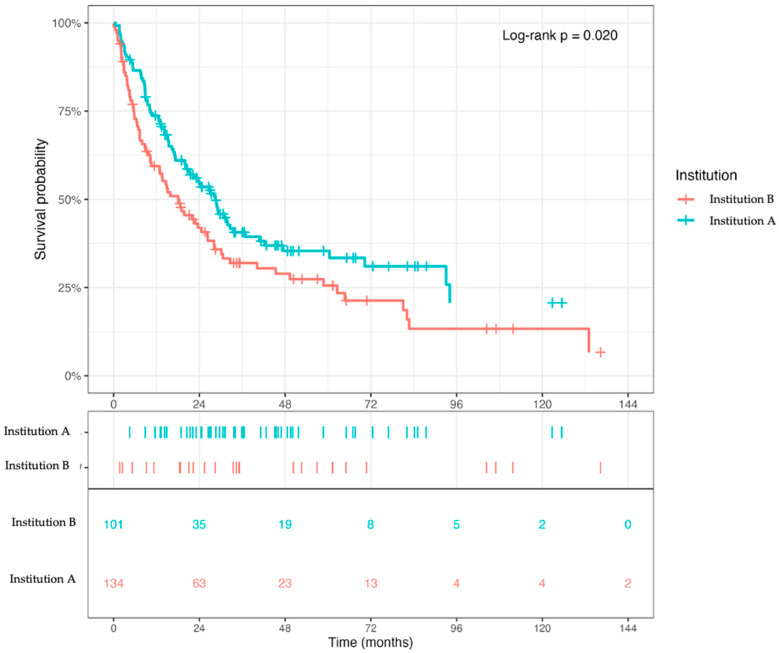
Post-metastasis survival by institution. Kaplan–Meier curves showing post-metastasis survival (PMS) for patients treated at Institution A and Institution B. PMS was defined from the first documentation of distant metastatic disease to death or last follow-up and was interpreted as descriptive institutional context for the metastatic phase.

As a second contextual survival analysis, the corresponding Kaplan–Meier curves for post-metastasis survival (PMS) suggested a numerical separation between the two contributing centers (Figure 3; Table 2). Median PMS was 18.1 months at Institution B and 28.5 months at Institution A. However, this comparison remained descriptive and did not establish whether the observed difference reflected an independent institutional effect or differences in disease course and prognostic composition. These center-stratified survival analyses were therefore interpreted as contextual descriptive analyses and not as evidence of comparative treatment effectiveness or institutional ranking.

### 3.3. Landmark-Based Prognostic Stratification by 12-Month Metastatic State

**Figure 4:**
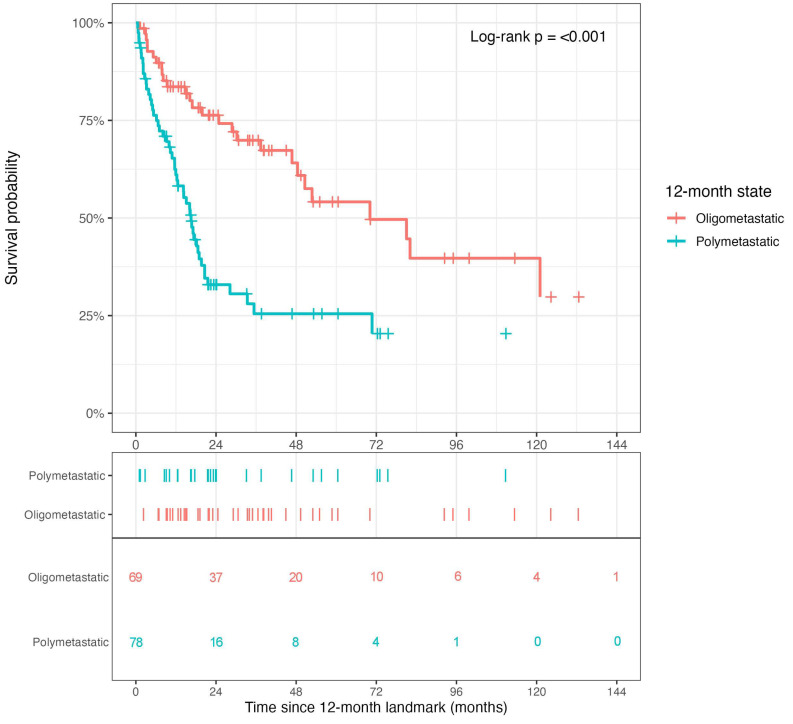
Landmark-based post-metastasis survival according to 12-month metastatic state. Kaplan–Meier curves showing survival from the 12-month landmark after first metastatic documentation, stratified by oligometastatic versus polymetastatic post-landmark burden state. Patients had to be alive and under observation at the landmark to be included.

To move beyond crude institutional comparison, a landmark-based prognostic stratification analysis was performed using realized metastatic state at 12 months. This analysis showed a clear post-landmark survival separation according to oligometastatic versus polymetastatic state at 12 months (Figure 4). Patients who remained oligometastatic at the 12-month landmark had substantially more favorable subsequent PMS than those who were polymetastatic, indicating that the landmark metastatic status is a strong prognostic discriminator after metastasis.

This pattern was consistent with the univariable Cox analyses (Table 3), in which state at the 12-month landmark emerged as the strongest single prognostic factor among the variables tested, whereas institution showed only a weaker and non-dominant association.

### 3.4. Multivariable Landmark Model

**Figure 5:**
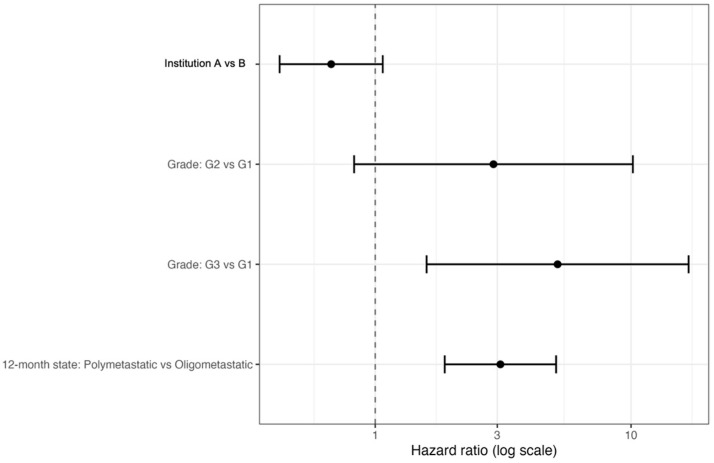
Multivariable landmark model for post-metastasis survival. Forest plot showing hazard ratios from the reduced multivariable Cox model for landmark PMS, including institution, histologic grade, and realized metastatic state at 12 months. The strongest adverse prognostic factor was polymetastatic post-landmark burden state, followed by high histologic grade.

The principal inferential analysis is presented in Figure 5 and Table 4. In the reduced multivariable landmark model, polymetastatic 12-month metastatic state remained the strongest independent predictor of inferior PMS (HR 3.09, 95% CI 1.87–5.09, *p* = 0.00001). High histologic grade also retained adverse prognostic significance, with G3 versus G1 associated with significantly worse PMS (HR 5.16, 95% CI 1.59–16.79, *p* = 0.006) and G2 versus G1 also showing an adverse association (HR 2.90, 95% CI 1.13–7.46, *p* = 0.027). By contrast, institution (Institution A vs. Institution B) showed only a non-significant trend after adjustment (HR 0.67, 95% CI 0.42–1.07, *p* = 0.094).

Taken together, these findings indicate that the apparent center-level signal is substantially attenuated once the analysis is anchored in the state at the 12-month landmark and tumor grade. In this cohort, outcome after metastasis was therefore explained more convincingly by disease aggressiveness and post-landmark burden state than by institution itself.

### 3.5. Integrated Interpretation

Across the analyses, the central pattern was consistent: institution-based survival curves provided descriptive context, whereas the strongest prognostic signal arose from the landmark-based analysis according to realized metastatic state at 12 months. In the multivariable landmark model, the landmark metastatic status and histologic grade outweighed institution as determinants of post-metastasis survival, supporting the relevance of a state-based interpretation of metastatic sarcoma evolution.

## 4. Discussion

This study addressed whether realized metastatic state at 12 months provides meaningful prognostic stratification for post-metastasis survival within a two-institution metastatic sarcoma cohort [9,21]. The main finding was that polymetastatic 12-month state was associated with substantially inferior post-metastasis survival and remained the dominant adverse factor in the landmark multivariable model, whereas the institutional signal became secondary after adjustment [8,15,22,23]. These results support the view that prognosis after metastatic onset is better understood through early metastatic evolution than through crude center attribution alone [11,13].

The clinical relevance of the 12-month landmark lies in its ability to capture not merely the presence of metastatic disease, but its early metastatic trajectory [24]. In this context, the distinction between persistent oligometastatic and polymetastatic disease at 12 months is likely to reflect more than a numerical difference in lesion count; rather, it may reflect underlying differences such as metastatic tempo, systemic aggressiveness, and residual capacity for local disease control [14,22,23]. This is biologically plausible in light of prior real-world-time observations showing that metastatic sarcoma phenotypes differ not only in burden, but also in terms of when metastases emerge and where they distribute, with lung-only and multi-organ pathways separating clinically meaningful dissemination architectures [11,13]. Patients who remain oligometastatic over this interval may represent a biologically more indolent subgroup in whom metastatic spread remains quantitatively or anatomically constrained, whereas progression to polymetastatic disease suggests broader systemic dissemination and loss of meaningful containment [14,25].

The oligo- versus polymetastatic distinction used here should also be interpreted critically. Although the present cutoff is clinically pragmatic and reproducible, it does not represent a universally validated sarcoma-specific biologic threshold. As in the broader oligometastatic literature, such definitions likely require disease-specific validation rather than simple transfer across all cancer types. The purpose of the current study was therefore not to establish a definitive sarcoma ontology of oligometastatic disease, but to test whether a reproducibly defined 12-month metastatic state provides prognostic value. At the same time, the selected landmark should not be overinterpreted as a uniquely validated sarcoma-specific threshold. A 12-month timepoint is, to some extent, a pragmatic choice, albeit one that is clinically defensible because it captures early metastatic evolution while preserving analytic interpretability. Whether other landmark timepoints may prove superior in specific histologic or therapeutic contexts remains an open question for future work.

A central methodological point of the present study is the distinction between OS and PMS, because the primary prognostic question addressed here lies within the metastatic phase of disease [21,26]. OS provides the full disease-course survival context, whereas PMS focuses specifically on survival after metastatic disease has emerged [20,27]. Because the state at the 12-month landmark is a metastatic-phase variable, it is more directly aligned with PMS than with OS [24]. In this sense, the landmark PMS model addresses a disease-proximal question: among patients who have entered the metastatic phase and remain alive and under observation at 12 months, does the post-landmark burden state meaningfully stratify subsequent outcome [28,29]? The present data suggest that it does [21,30]. This likely explains why the signal is strongest when the analysis is anchored in PMS rather than in the full OS trajectory [7,13,26].

An equally important implication of the present analysis is that the observed crude survival differences between institutions should be interpreted with caution [17]. Although PMS was numerically longer at one institution in the unadjusted Kaplan–Meier analysis, this pattern was not retained as a dominant independent effect once the model accounted for state at the 12-month landmark and histologic grade [31]. This suggests that the initial center-level separation is more plausibly understood as a descriptive signal arising from differences in prognostic composition, disease evolution, referral pathways, or other unmeasured aspects of case-mix, rather than as direct evidence of a center effect in itself [32]. In observational multi-institutional sarcoma datasets, such attenuation after adjustment is particularly important, because institutional comparisons are inherently vulnerable to confounding by indication, selection mechanisms, and differences in timing of referral or metastasis-directed interventions [33]. The present findings therefore do not support a strong conclusion that institutional affiliation independently determines outcome, nor should they be interpreted as evidence for differential treatment effectiveness between centers [34].

From a clinical perspective, these findings are relevant because they support the idea that oligometastatic persistence is not merely a descriptive category, but a potentially meaningful prognostic state [14]. If patients who remain oligometastatic at 12 months experience substantially better subsequent survival, this suggests that early metastatic evolution may help identify a subgroup with a more indolent course and, potentially, a greater window for sustained benefit from metastasis-directed or locally consolidative strategies [35]. Conversely, transition to polymetastatic disease within the same interval appears to mark a more aggressive systemic phenotype, for which prognosis is driven less by local control and more by disseminated biologic behavior [22]. Read together with prior data showing that sarcoma metastases cluster along distinct temporal and organ-tropism pathways, the present findings suggest that prognostic assessment and follow-up strategy should increasingly account for how metastatic phenotype evolves, not merely whether metastases are present [13,36].

This interpretation should also be viewed in the context of treatment heterogeneity. In metastatic sarcoma, outcome after metastatic onset may be shaped not only by disease biology and metastatic state, but also by systemic therapy, metastasis-directed surgery, radiotherapy, ablative procedures, and individualized multidisciplinary decision-making. The present study was not designed to estimate comparative treatment effects and therefore should not be interpreted as a therapeutic effectiveness analysis. Rather, it provides a prognostic framework within which future treatment-linked and histology-aware analyses may be more meaningfully structured.

Beyond prognostic counseling, the post-landmark burden state may also help identify a subgroup with particularly adverse post-metastatic outcome for whom intensified reassessment, closer multidisciplinary review, or inclusion in risk-enriched therapeutic studies may be appropriate [37,38]. However, the present analysis is prognostic rather than predictive and does not establish that patients with polymetastatic landmark metastatic status derive greater benefit from systemic treatment [39]. Likewise, the present study was not designed to compare chemotherapy use, sequencing, or duration between centers. Whether this variable has predictive utility for chemotherapy benefit or other systemic strategies would require dedicated treatment-linked analysis [40]. This distinction is important, because the preceding triangulation work provided a biologic rationale for stratified therapeutic frameworks, but did not evaluate survival or treatment efficacy directly [13].

### Limitations

This study has several limitations. First, this was a retrospective observational analysis of a two-institution cohort, and the inherent constraints of non-randomized real-world data remain operative despite structured modeling [19]. Institutional comparisons are particularly susceptible to referral bias, treatment-selection effects, and unmeasured differences in case-mix [41].

Second, although the landmark design improves on a crude baseline metastatic yes/no distinction by incorporating realized early metastatic course up to 12 months, it also restricts the analysis to patients surviving and remaining under observation until that landmark [42]. Moreover, the present framework does not account for post-landmark time-varying systemic therapy, metastasis-directed interventions, repeated metastatic transitions, or evolving patient health status. Accordingly, the analysis should be interpreted as a clinically structured prognostic framework rather than as a fully dynamic longitudinal survival model.

Third, the cohort is histologically heterogeneous, which reflects the biological breadth of metastatic sarcoma but also limits the granularity of subtype-specific inference. The present landmark cohort was not sufficiently powered to support robust histology-specific subgroup analyses across the major subtypes without risking unstable inference. We therefore chose not to present potentially underpowered subtype-specific results and instead interpret the findings as applying to a heterogeneous metastatic sarcoma population that will require validation in larger histology-aware datasets.

Fourth, the present 12-month metastatic state definition captures metastatic burden in a pragmatic operational sense, but it does not represent the full multidimensional severity architecture of metastatic disease. Features such as metastatic volume, brain or other critical visceral involvement, pathologic fracture, neurologic compromise, and symptom burden may also carry prognostic relevance but were not sufficiently standardized across the cohort for formal inclusion. The current results should therefore be interpreted as applying to 12-month metastatic burden state rather than to all biologic and clinical dimensions of metastatic severity.

Fifth, important treatment-related determinants of post-metastatic survival were not fully modeled. These include systemic therapy exposure, treatment sequencing and duration, metastasis-directed surgery, radiotherapy, ablative approaches, and local consolidative treatment intent. Accordingly, the present study should be interpreted as prognostic rather than predictive and should not be read as evidence of comparative treatment effectiveness between institutions or therapeutic strategies [5].

Finally, while the preceding triangulation analysis and the current study are conceptually aligned, they address different analytic questions [13]. The former focused on metastatic phenotype architecture, whereas the present study examined the prognostic consequence of realized metastatic state at 12 months. These analyses are complementary but not interchangeable.

Despite these limitations, the present study remains clinically informative because it shows that prognosis within the metastatic phase of sarcoma is not adequately captured by crude institutional attribution or by a static baseline metastatic yes/no dichotomy alone. Instead, the realized metastatic state at 12 months provided a more informative framework for subsequent post-metastasis survival. In this sense, the study offers a practical intermediate step toward more biologically and temporally grounded metastatic risk assessment, even though treatment-linked and fully dynamic validation will require further work.

## 5. Conclusions

This two-institution analysis indicates that prognostic assessment in metastatic sarcoma may be strengthened by moving beyond crude center attribution toward a longitudinal state-based prognostic framework [43]. The most informative signal in this cohort was 12-month metastatic state, which outperformed institution as an explanatory variable after adjustment and remained closely aligned with histologic grade as a marker of biologic aggressiveness [44]. These findings support the concept that survival after metastatic progression is shaped predominantly by disease biology and metastatic-state evolution over time, rather than by institutional attribution alone [20]. Future work should therefore focus on validating the post-landmark burden state in larger, histology-aware datasets and on determining whether this variable can be integrated with treatment exposure and baseline risk tools to refine individualized metastatic sarcoma management [45].

## Figures and Tables

**Table 1:** Baseline characteristics of the metastatic sarcoma cohort overall and by institution. Baseline demographic and clinicopathologic variables are shown for the full cohort and stratified by Institution A and Institution B. Values are presented as counts and percentages for categorical variables and as summary statistics for continuous variables, as appropriate.

Variable	Level	Overall	Institution A	Institution B	*p* Value
Institution	Institution B	101 (42.8%)	0 (0.0%)	101 (100.0%)	<0.001
	Institution A	135 (57.2%)	135 (100.0%)	0 (0.0%)	
Age at diagnosis, years		59.0 [47.0, 72.0]	57.0 [45.0, 72.0]	64.0 [48.0, 72.0]	0.042
Gender	Female	118 (50.0%)	65 (48.1%)	53 (52.5%)	0.599
	Male	118 (50.0%)	70 (51.9%)	48 (47.5%)	
Histology group	Synovial sarcoma	7 (3.0%)	5 (3.7%)	2 (2.0%)	0.665
	Undifferentiated/unclassified sarcoma	40 (16.9%)	26 (19.3%)	14 (13.9%)	
	Conventional osteosarcoma	7 (3.0%)	5 (3.7%)	2 (2.0%)	
	Ewing sarcoma	9 (3.8%)	6 (4.4%)	3 (3.0%)	
	Dedifferentiated liposarcoma	18 (7.6%)	12 (8.9%)	6 (5.9%)	
	Leiomyosarcoma (excluding skin)	42 (17.8%)	22 (16.3%)	20 (19.8%)	
	Angiosarcoma of soft tissues	16 (6.8%)	7 (5.2%)	9 (8.9%)	
	Other	97 (41.1%)	52 (38.5%)	45 (44.6%)	
Grade	G1	14 (6.1%)	7 (5.3%)	7 (7.4%)	0.418
	G2	43 (18.9%)	22 (16.5%)	21 (22.1%)	
	G3	171 (75.0%)	104 (78.2%)	67 (70.5%)	
Tumor size, mm		95.0 [62.5, 135.0]	90.0 [61.5, 135.0]	100.0 [65.0, 135.0]	0.795
Primary site group	Pelvis combinations	8 (3.4%)	4 (3.0%)	4 (4.0%)	0.404
	Uterus	14 (5.9%)	6 (4.4%)	8 (7.9%)	
	anterior/extensor compartment: combinations	5 (2.1%)	5 (3.7%)	0 (0.0%)	
	Intraperitoneal	20 (8.5%)	10 (7.4%)	10 (9.9%)	
	Retro-/extraperitoneal	29 (12.3%)	17 (12.6%)	12 (11.9%)	
	Lung	6 (2.5%)	2 (1.5%)	4 (4.0%)	
	Head	6 (2.5%)	4 (3.0%)	2 (2.0%)	
	Other	148 (62.7%)	87 (64.4%)	61 (60.4%)	
Baseline metastatic	No	145 (61.7%)	83 (61.9%)	62 (61.4%)	0.156
	Unknown	3 (1.3%)	0 (0.0%)	3 (3.0%)	
	Yes	87 (37.0%)	51 (38.1%)	36 (35.6%)	
Metastasis-free interval, months		5.2 [0.0, 25.0]	4.2 [0.0, 18.3]	8.0 [0.0, 32.0]	0.063
Synchronicity	Metachronous	111 (47.0%)	59 (43.7%)	52 (51.5%)	0.292
	Synchronous	125 (53.0%)	76 (56.3%)	49 (48.5%)	
12-month metastatic state	Oligometastatic	94 (40.0%)	55 (41.0%)	39 (38.6%)	0.809
	Polymetastatic	141 (60.0%)	79 (59.0%)	62 (61.4%)	

**Table 2:** Summary of overall survival and post-metastasis survival by institution. Median survival estimates for overall survival (OS) and post-metastasis survival (PMS) are shown for Institution A and Institution B. These results provide descriptive center-level context for the subsequent landmark-based prognostic analyses.

Endpoint	Group	*n*	Events	Median Survival, Months [95% CI]
**OS**				
	Institution B	101	74	43.0 (21.9–71.1)
	Institution A	135	81	44.8 (33.2–76.0)
**PMS**				
	Institution B	101	74	18.1 (12.9–26.3)
	Institution A	135	81	28.5 (21.2–36.8)

**Table 3:** Univariable Cox models for landmark post-metastasis survival. Univariable hazard ratios, 95% confidence intervals, and *p*-values are shown for candidate prognostic variables tested in relation to landmark PMS. This table provides the unadjusted associations supporting the multivariable landmark model.

Model	Term	*n*	Events	HR	Lower 95% CI	Upper 95% CI	*p*
Institution	Institution A	147	78	0.81	0.51	1.27	0.36
Grade	Grade G2	147	78	2.62	0.75	9.10	0.12
Grade	Grade G3	147	78	4.53	1.39	14.66	0.011
Status	Polymetastatic	147	78	2.78	1.71	4.51	<0.001

**Table 4:** Reduced multivariable Cox model for landmark post-metastasis survival. Hazard ratios, 95% confidence intervals, and *p*-values are shown for the reduced multivariable Cox regression model assessing landmark PMS from the 12-month landmark, including institution, histologic grade, and 12-month metastatic state.

Model	Term	*n*	Events	HR	Lower 95% CI	Upper 95% CI	*p*
PMS 12 m	Institution A	147	78	0.67	0.42	1.07	0.09
PMS 12 m	Grade G2	147	78	2.899	0.827	10.16	0.09
PMS 12 m	Grade G3	147	78	5.16	1.58	16.789	0.006
PMS 12 m	Polymetastatic	147	78	3.08	1.86	5.09	<0.001

## Data Availability

The data presented in this study are available on request from the corresponding author.

## Supplementary Materials

The following supporting information can be downloaded at: https://www.mdpi.com/article/10.3390/cancers18101622/s1, Figure S1. Overall survival by AJCC broad stage; Table S1: Additional multivariable Cox model for overall survival including AJCC broad stage.
