# Supplementary material for: Twelve-Month Metastatic State as a Landmark-Based Prognostic Stratifier in Metastatic Sarcoma
Source: Cancers (Basel). 2026 May 17;18(10):1622. doi: 10.3390/cancers18101622 (PMC13204580; doi:10.3390/cancers18101622)
Supplement: Supplementary file 1 [file cancers-18-01622-s001.zip › cancers-4285029-supplementary.pdf]

.

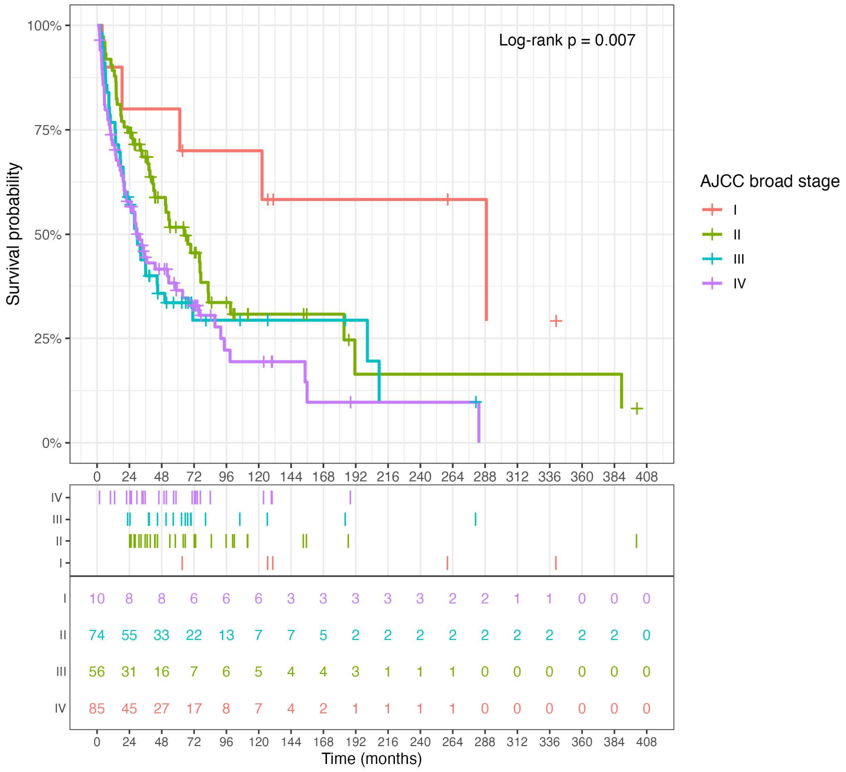


**Figure S1. Overall survival by AJCC broad stage.** Kaplan-Meier curves showing overall survival (OS) according to AJCC broad stage. This analysis is presented as contextual validation of the clinical coherence of the cohort and not as the principal prognostic analysis.

**Table S1. Additional multivariable Cox model for overall survival including AJCC broad stage.** Hazard ratios, 95% confidence intervals, and p-values are shown for the supportive multivariable OS model including AJCC broad stage and selected clinicopathologic variables. This analysis is presented as contextual validation rather than the primary prognostic model.

| Model | Term | n | Events | HR | Lower 95% CI | Upper 95% CI | p |
|---|---|---|---|---|---|---|---|
| OS multivariable | Institution A | 218 | 144 | 0.73 | 0.52 | 1.02 | 0.06 |
| OS multivariable | Grade G2 | 218 | 144 | 2.32 | 0.77 | 6.93 | 0.13 |
| OS multivariable | Grade G3 | 218 | 144 | 4.22 | 1.499 | 11.90 | 0.006 |
| OS multivariable | AJCC II | 218 | 144 | 1.45 | 0.50 | 4.23 | 0.49 |
| OS multivariable | AJCC II | 218 | 144 | 2.27 | 0.77 | 6.669 | 0.13 |
| OS multivariable | AJCC IV | 218 | 144 | 2.36 | 0.82 | 6.78 | 0.10 |
